# The prognostic role of cigarette smoking in Kidney Cancer Survival

**DOI:** 10.1002/cam4.6104

**Published:** 2023-05-18

**Authors:** Amrit Baral, Hannah M. Cranford, Janaki Sharma, Paulo S. Pinheiro

**Affiliations:** ^1^ Department of Public Health Sciences University of Miami Miller School of Medicine Florida USA; ^2^ Sylvester Comprehensive Cancer Center University of Miami Miller School of Medicine Florida USA

**Keywords:** histology types, kidney cancer, prognosis, smoking, survival

## Abstract

**Background:**

The role of cigarette smoking as a prognostic factor for kidney cancer (KC) is unclear. In this population‐based study, we analyze cancer‐specific survival (CSS) outcomes among KC patients by smoking status at diagnosis in the diverse state of Florida.

**Methods:**

All primary KC cases from the Florida Cancer Registry diagnosed during 2005–2018 were analyzed. Cox proportional regression was conducted to assess the determinants of KC survival, including age, sex, race/ethnicity, socioeconomic status, histology type, cancer stage, and treatment received with a particular focus on smoking status (smokers at diagnosis referred to as current smokers, former smokers, and never smokers).

**Results:**

Among all 36,150 KC patients, 18.3% were smokers at diagnosis (*n* = 6629), 32.9% were former smokers (*n* = 11,870), and 48.8% were never smokers (*n* = 17,651). Age‐standardized five‐year survival for current, former, and never smokers was 65.3 (95% CI: 64.1–66.5), 70.6 (95% CI: 69.7–71.5), and 75.3 (95% CI: 74.6–76.0) respectively. In multivariable analysis, current and former smokers had an estimated 30% and 14% higher risk of KC death compared to never smokers, respectively, after adjusting for potential confounders (HR: 1.30, 95% CI: 1.23–1.40; HR: 1.14, 95% CI: 1.10–1.20).

**Conclusion:**

Smoking independently contributes to poorer survival, across all KC stages. Clinicians should encourage and facilitate participation in cigarette smoking cessation programs targeted at current smokers. Prospective studies are warranted to assess the role of different types of tobacco use and cessation programs on KC survival.

## 1 INTRODUCTION

Kidney cancer (KC) is the ninth most common neoplasm in the United States of America (US),^1^ with an estimated 76,080 new cases and 13,780 deaths from kidney and renal pelvis cancers in 2021.^2^ Incidence rates of KC are rising globally, primarily attributed to the early detection and inadvertent discovery of early‐stage tumors with the widespread use of advanced imaging diagnostics such as Computed Tomography and Magnetic Resonance Imaging.^1^ Although KC mortality from metastatic disease is decreasing due to improved diagnostic techniques and treatments, specifically following the discovery of anti‐angiogenic and immune therapies.^1^, ^3^ KC in adults can be broadly divided into two main types: renal cell carcinoma (RCC), arising from the renal cortex, and renal transitional cell carcinoma (RTCC), originating from the renal pelvis.^4^ RCC represents 80%–85% of KC and RTCC, 8%.^5^, ^6^ RCC is further classified into major histologic types: clear cell, papillary, and chromophobe, among others.^7^, ^8^

Cigarette smoking is an established risk factor for KC among others including obesity, chronic renal disease, toxic chemical exposure, certain medications, and hypertension.^9^ As compared to never smokers, current and former smokers have an increased incidence of KC.^10^ However, the prognostic role of smoking in KC is unclear. A few studies have shown that smoking is associated with poor survival and may increase the mortality risk in patients with RCC, but findings have been inconsistent,^11^, ^12^, ^13^ often based on overall survival (OS) and restricted to subsets of specifically treated patients such as metastatic RCC receiving cytoreductive nephrectomy (CNT).^11^, ^14^ Moreover, these studies were conducted before the introduction of newer treatment modalities such as immune checkpoint inhibitors or combination therapy with tyrosine kinase inhibitors (TKI) and were based on small sample sizes in hospital settings.^15^, ^16^ Thus far, an all‐inclusive study, encompassing all stages of disease from a diverse population‐based study, does not exist.

In this study, we study the role of smoking status, current, former or never smoker on Kidney Cancer Survival using individual level data from the Florida Cancer Data System (FCDS), the statewide cancer registry.

## 2 MATERIALS AND METHODS

Data for all cases of first primary KC in Florida, aged 15 and older, during 2005–2018 were obtained from FCDS. FCDS has met or exceeded the North American Association of Central Cancer Registries (NAACCR) standards of quality, timeliness, and completeness (>95%) for every year since 1995.^17^, ^18^ Cases of primary KC site codes (C64.9 and C65.9) and morphology codes according to the International Classification of Diseases for Oncology, third edition (ICD‐O‐3) were included.^19^ Sociodemographic variables comprised age, sex, race/ethnicity, socioeconomic status, and insurance type. Clinical‐pathological factors including smoking status, histology, tumor stage, and treatment (surgery, chemotherapy, and radiation) (Table 1), as well as the date and cause of death were included.

**TABLE 1:** Population characteristics and clinical features of kidney cancer by smoking status at diagnosis, Florida 2005–2018.

Characteristics/categories	Overall *N* (%)	Never smokers *n* (%)	Ever smokers *n* (%)	*p* value^a^
Total (*N*)	36,150	17,651 (48.8)	18,499 (51.2)	
Age at diagnosis (years)				<0.0001
15–44	2360 (6.5)	1335 (56.6)	1025 (43.4)	
45–54	4886 (13.5)	2425 (49.6)	2461 (50.4)	
55–64	8525 (23.6)	3937 (46.2)	4588 (53.8)	
65–74	10,833 (30.0)	4979 (46.0)	5854 (31.6)	
75+	9546 (26.4)	4975 (52.1)	4571 (47.9)	
Median age (years)	67	66	67	0.443
Sex				<0.0001
Male	22,831 (63.2)	9823 (43.0)	13,008 (57.0)	
Female	13,319 (36.8)	7828 (58.8)	5491 (41.2)	
Race/Ethnicity				<0.0001
NHW	26,755 (74.0)	12,112 (45.3)	14,643 (54.7)	
NHB	3751 (10.4)	2117 (56.4)	1634 (43.6)	
American Indian	44 (0.1)	20 (45.4)	24 (54.6)	
API	327 (0.9)	203 (62.1)	124 (37.9)	
Hispanic	4996 (13.8)	3030 (60.6)	1966 (39.4)	
Mixed/Other	277 (0.8)	169 (61.0)	108 (39.0)	
Socioeconomic status				0.045
High	4464 (12.3)	2232 (50)	2232(50)	
Medium	10,502 (29.0)	5086 (48.4)	5416 (51.6)	
Low	13,235 (36.7)	6373 (48.1)	6862 (51.9)	
Very low	7646 (21.1)	3818 (49.9)	3828 (50.1)	
Unknown	303 (0.9)	142 (46.9)	161 (53.1)	
Insurance				<0.0001
Private	14,865 (41.1)	7686 (51.7)	7179 (48.3)	
Medicare	14,776 (40.9)	6931 (46.9)	7845 (53.1)	
Medicaid	3006 (8.3)	1385 (46.1)	1621 (53.9)	
No insurance	1280 (3.5)	525 (41.0)	755 (59.0)	
Unknown	2223 (6.2)	1124 (50.6)	1099 (49.4)	
Histology				<0.0001
RCC NOS	11,154 (30.8)	5687 (51.0)	5467 (49.0)	
Chromophobe	1444 (4.0)	869 (60.2)	575 (38.8)	
Clear cell	14,592(40.4)	7138 (48.9)	7454 (51.1)	
Papillary	3759 (10.4)	1789 (47.6)	1970 (52.4)	
RTCC	2799 (7.8)	1048 (37.4)	1751 (62.6)	
Other	2402 (6.6)	1120 (46.6)	1282 (53.4)	
Stage				<0.0001
Localized	23,472 (64.9)	11,804 (50.3)	11,668 (49.7)	
Regional	5386 (14.9)	2506 (46.5)	2880 (53.5)	
Distant	5289 (14.6)	2322 (43.9)	2967 (56.1)	
Unknown	2003 (5.6)	1019 (50.8)	984 (49.1)	
Surgery				0.006
Yes	28,239 (78.1)	13,913 (49.3)	14,326 (50.7)	
No	7838 (21.7)	3702 (47.2)	4136 (52.8)	
Unknown	73 (0.2)	36 (49.3)	37 (50.7)	
Chemotherapy				<0.0001
Yes	2953 (8.2)	1220 (41.3)	1733 (58.7)	
No	32,868 (90.9)	16,307 (49.6)	16,561 (50.4)	
Unknown	329 (0.9)	124 (39.7)	205 (62.3)	
Radiation				<0.0001
Yes	1297 (3.6)	547 (42.2)	750 (57.8)	
No	34,755 (96.1)	17,061 (49.1)	17,694 (50.9)	
Unknown	98 (0.3)	43 (43.9)	55 (56.1)	
Primary tumor location				<0.0001
Kidney proper (renal cortex, C64.9)	33,528 (92.7)	16,665 (49.7)	16,863 (50.3)	
Renal pelvis (C65.9)	2522 (7.3)	986 (37.6)	1636 (62.4)	

Abbreviations: APIs, Asian and Pacific Islanders; NHW, non‐Hispanic White; NHB, Non‐Hispanic Black; RCCNOS, renal cell carcinoma not otherwise specified; RTCC, renal transitional cell carcinoma.

^a^ *p* values were calculated using chi‐squared test and Student's *t*‐test where appropriate.

Histology was classified according to previous research^4^: chromophobe (8270,8317), clear cell (8005,8310), papillary (8260), renal cell carcinoma not otherwise specified (RCC NOS) (8312), renal transitional cell carcinoma (RTCC) (8050,8070,8071,8074,8082,8120,8122, 8130,8131), and others (8000‐ 8004,8010‐8046,8140‐ 8290,8311‐8323,8480‐ 8714). The Surveillance, Epidemiology, and End Results (SEER) staging categories (localized, regional, distant, and unknown) were used to define the KC stage at diagnosis. FCDS smoking status was categorized as never smokers (having consumed less than 100 cigarettes in their lifetime) and ever smokers (including current and former). FCDS Data completeness for cigarette smoking status collected since the year 2000 is over 80%. The proportion of the population living under the poverty level in the census tract of residence was the basis for classifying socio‐economic status. Those individuals in tracts 0% to <5%, 5% to <10%, 10% to <20%, and 20% to <100% were categorized as very low poverty level, low, intermediate, high, and unknown poverty level respectively.^20^ The type of insurance was classified as Medicaid, Medicare, private, no insurance, and unknown. Race‐ethnicity was classified into mutually exclusive groups as non‐Hispanic White (White), non‐Hispanic Black (Black), non‐Hispanic Asian and Pacific Islander (API), American Indians, and Hispanics of any race. Carcinoma in situ, benign tumors, sarcomas, other rare histology types, and childhood tumors were excluded from the analysis. Cases with unknown smoking status at diagnosis represented 20.6% of the total cases and were excluded from the analysis.

### 2.1 Statistical analysis

The distributions of demographic variables, tumor characteristics, and treatment variables by smoking status at diagnosis were compared and tested using the Chi‐squared test and Student's *t*‐test where appropriate (Table 1).

**FIGURE 1:**
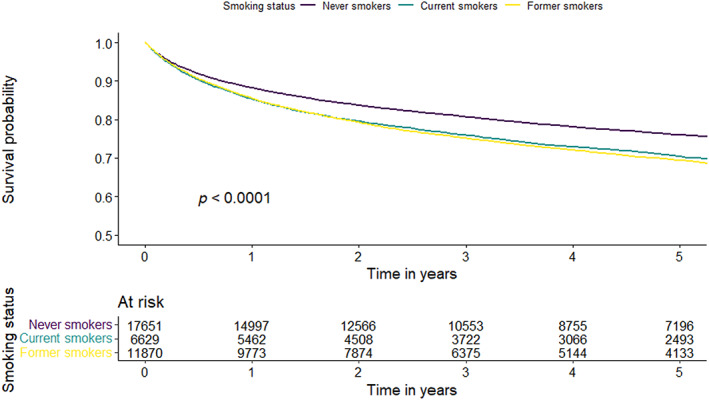
Kaplan–Meier estimates of kidney cancer‐specific survival by smoking status at diagnosis.

**FIGURE 2:**
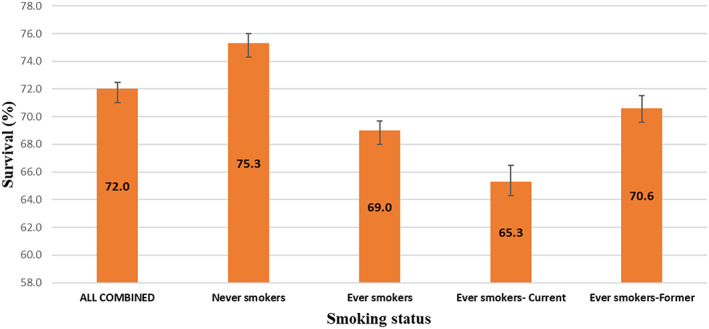
Age‐standardized five (5)‐year kidney cancer‐specific survival estimates with 95% CI by smoking status at diagnosis using the life‐table method.

The study outcome was KC‐specific mortality based on cause of death information obtained from the death certificate and following specific SEER rules for cause‐specific cancer death for KC as a first primary.^21^ The observed survival time was calculated as the difference between the date of diagnosis and the date of death due to KC or 31 December 2018, whichever occurred first. Patients who died of other causes or were alive at the end of the study period (December 31, 2018) were censored. To examine differences in CSS among smoking categories (former, current, and never smokers) at diagnosis, Kaplan Meier survival curves and the log‐rank test were used (Figure 1). Five‐year population‐based CSS was calculated using the lifetable method for the entire population and by two and three levels of smoking (Figure 2), adjusting for age according to the International Cancer Survival Standards.^22^ Cox proportional hazards regression analysis was used to assess potential predictors of CSS in both univariable and multivariable models including age, sex, socioeconomic status, race/ethnicity, smoking status at diagnosis (never, ever, and former), insurance type, tumor histological type, stage, and treatment (surgery, chemotherapy, and radiation) (Table 2).

**TABLE 2:** Univariable and multivariable Cox regression analysis assessing predictors of kidney cancer mortality, Florida 2005–2018.

Prognostic factors/categories	Univariable analysis			Multivariable analysis		
	HR	95% CI	*p* value	HR	95% CI	*p* value
Smoking status						
Never (Ref)	‐	‐	‐	‐	‐	
Former	1.34	1.28–1.40	<0.0001	1.14	1.10–1.20	<0.0001
Current	1.29	1.22–1.36	<0.0001	1.30	1.23–1.40	<0.0001
Age (years)						
15–44 (Ref)	‐	‐	‐	‐	‐	
45–54	1.63	1.43–1.86	<0.0001	1.40	1.22–1.60	<0.0001
55–64	2.26	2.00–2.56	<0.0001	1.73	1.53–1.96	<0.0001
65–74	2.75	2.43–3.10	<0.0001	2.12	1.87–2.40	<0.0001
75+	4.5	4.0–5.08	<0.0001	2.92	2.57–3.32	<0.0001
Sex						
Female (Ref)	‐	‐	‐	‐	‐	
Male	1.17	1.12–1.22	<0.0001	1.10	1.06–1.15	<0.0001
Race/ethnicity						
NHW (Ref)	‐	‐	‐	‐	‐	
NHB	0.86	0.80–0.92	<0.0001	1.00	0.93–1.07	0.99
American Indians	1.20	0.71–2.03	0.49	1.08	0.64–1.2	0.77
APIs	1.10	0.90–1.35	0.32	1.04	0.85–1.27	0.73
Hispanics	0.88	0.83–0.93	<0.0001	0.82	0.77–0.87	<0.0001
Mixed/Others	0.24	0.16–0.38	<0.0001	0.30	0.20–0.47	<0.0001
Socioeconomic status						
High (Ref)	‐	‐	‐	‐	‐	
Medium	1.09	1.02–1.17	0.008	0.99	0.93–1.06	0.80
Low	1.15	1.08–1.23	<0.0001	1.07	1.00–1.14	0.04
Very Low	1.12	1.04–1.20	0.001	1.07	0.99–1.15	0.07
Unknown	1.14	0.93–1.40	0.204	1.03	0.84–1.27	0.09
Insurance						
Medicare (Ref)	‐	‐	‐	‐	‐	
Medicaid	0.99	0.92–1.06	0.772	1.15	1.06–1.24	0.0004
Private	0.65	0.62–0.68	<0.0001	0.93	0.89–0.98	0.0044
No Insurance	0.82	0.74–0.92	0.0004	0.96	0.86–1.08	0.483
Unknown	0.75	0.69–0.82	<0.0001	0.95	0.87–1.04	0.250
Histology						
Clear cell (Ref)	‐	‐	‐	‐	‐	
Papillary	0.80	0.73–0.87	<0.0001	1.02	0.93–1.11	0.64
RCC NOS	1.94	1.85–2.04	<0.0001	1.30	1.23–1.36	<0.0001
Chromophobe	0.44	0.37–0.52	<0.0001	0.61	0.51–0.72	<0.0001
RTCC	3.23	3.03–3.45	<0.0001	1.80	1.69–1.93	<0.0001
Others	3.50	3.28–3.75	<0.0001	2.01	1.88–2.16	<0.001
Stage						
Localized (Ref)	‐	‐	‐	‐	‐	
Regional	3.77	3.56–4.0	<0.0001	3.44	3.24–3.65	<0.0001
Distant	16.75	15.96–17.60	<0.0001	9.91	9.33–10.53	<0.0001
Unknown	3.82	3.54–4.14	<0.0001	1.85	1.70–2.02	<0.0001
Surgery						
Yes (Ref)	‐	‐	‐	‐	‐	
No	5.57	5.35–5.80	<0.0001	2.40	2.27–2.51	<0.0001
Unknown	3.25	2.35–4.49	<0.0001	1.66	1.20–2.30	<0.01
Chemotherapy						
Yes (Ref)	‐	‐	‐	‐	‐	
No	0.19	0.18–0.20	<0.0001	1.0	0.95–1.06	0.94
Unknown	0.94	0.81–1.08	0.33	1.17	1.02–1.34	0.03
Radiation						
Yes (Ref)	‐	‐	‐	‐	‐	
No	0.17	0.16–0.18	<0.0001	0.85	0.79–0.91	<0.0001
Unknown	0.52	0.40–0.70	<0.0001	0.79	0.60–1.05	0.10

Abbreviations: APIs, Asian and Pacific Islanders; CI, confidence interval; HR, hazard ratio; NHB, non‐Hispanic Black; NHW, non‐Hispanic White; RCCNOS, renal cell carcinoma not otherwise specified; RTCC, renal transitional cell carcinoma.

To directly assess the prognostic role of cigarette smoking, we directly compared current smokers with former smokers in a model restricted to ever smokers. The rationale for this subset analysis is that smoking is likely associated with important unmeasured comorbidities for both current and past/former smokers that impact KC survival. These important comorbidities may include heart disease, chronic obstructive pulmonary disease, hypertension, and cerebrovascular disease among others.^23^, ^24^ Thus, a comparison between current and former smokers is of value as in addition to the smoking‐related comorbidities potentially existing in both groups of ever smokers, there is the possible additional independent prognostic effect of current smoking not found among former smokers. Moreover, to investigate this independent effect across all stages of the disease, we built Cox regression models directly comparing current to former smokers, the reference category, for all stages combined, and stratified for distant stage, and for the localized and regional stages (Table 3). The proportional hazard assumption was met, supported both graphically and by Schoenfeld residual tests. All tests were two‐sided with an alpha level of 0.05 and statistical software SAS 9.4 was used for analyses.

**TABLE 3:** Multivariable Cox regression analysis models assessing predictors of kidney cancer mortality by smoking status and cancer stage, Florida 2005–2018.

Prognostic factors/categories	Model 1			Model 2			Model 3			Model 4		
	Current vs. former smokers (All stages)			Current vs. former smokers (local‐regional stages only)			Current vs. former smoker (distant stage only)			Current vs. former smokers (distant stage only)^a^		
	HR	95% CI	*p* value	HR	95% CI	*p* value	HR	95% CI	*p* value	HR	95% CI	*p* value
Smoking status												
Former (Ref)	‐	‐	‐	‐	‐	‐	‐	‐	‐	‐	‐	‐
Current	1.13	1.06–1.20	<0.0001	1.21	1.12–1.32	<0.0001	1.07	0.99–1.17	0.0985	1.10	1.01–1.20	0.0284
Age (years)												
15–44 (Ref)	‐	‐	‐	‐	‐	‐	‐	‐	‐	‐	‐	‐
45–54	1.44	1.19–1.74	0.0002	2.62	1.84–3.74	<0.0001	0.93	0.78–1.10	0.4070	0.93	0.78–1.10	0.3779
55–64	1.83	1.53–2.20	<0.0001	4.06	2.88–5.72	<0.0001	0.94	0.80–1.10	0.4521	0.95	0.81–1.11	0.5254
65–74	2.26	1.88–2.72	<0.0001	5.23	3.70–7.38	<0.0001	1.09	0.92–1.28	0.3226	1.08	0.93–1.27	0.3068
75+	2.96	2.45–3.57	<0.0001	7.63	5.34–10.80	<0.0001	1.26	1.07–1.49	0.0066	1.26	1.08–1.47	0.0042
Sex												
Female (Ref)	‐	‐	‐	‐	‐	‐	‐	‐	‐	‐	‐	‐
Male	1.12	1.06–1.19	0.0002	1.17	1.10–1.28	<0.0001	1.03	0.96–1.10	0.3803	‐	‐	‐
Race/ethnicity												
NHW (Ref)	‐	‐	‐	‐	‐	‐	‐	‐	‐	‐	‐	‐
NHB	1.05	0.95–1.16	0.3525	1.06	0.92–1.22	0.4477	0.99	0.88–1.10	0.7963	‐	‐	‐
American Indians	1.26	0.70–2.27	0.4522	2.50	1.11–5.53	0.0264	1.18	0.53–2.63	0.6926	‐	‐	‐
APIs	1.12	0.82–1.53	0.4674	1.10	0.70–1.75	0.6549	0.95	0.69–1.30	0.7519	‐	‐	‐
Hispanic	0.86	0.78–0.94	0.0009	0.89	0.78–1.01	0.0835	0.76	0.69–0.84	<0.0001	‐	‐	‐
Mixed/Others	0.42	0.23–0.76	0.0041	0.15	0.04–0.60	0.0072	0.57	0.32–1.01	<0.0001	‐	‐	‐
Socioeconomic status												
High (Ref)	‐	‐	‐	‐	‐	‐	‐	‐	‐	‐	‐	‐
Medium	0.91	0.83–1.00	0.0504	0.95	0.84–1.07	0.3927	0.98	0.88–1.09	0.6781	‐	‐	‐
Low	0.98	0.90–1.07	0.6344	1.02	0.91–1.15	0.7390	1.02	0.92–1.14	0.6443	‐	‐	‐
Very Low	1.00	0.91–1.11	0.8983	1.02	0.89–1.18	0.7298	1.02	0.91–1.15	0.6855	‐	‐	‐
Unknown	0.90	0.69–1.18	0.4549	1.09	0.72–1.63	0.6860	0.92	0.67–1.26	0.6005	‐	‐	‐
Insurance												
Medicare (Ref)	‐	‐	‐	‐	‐	‐	‐	‐	‐	‐	‐	‐
Medicaid	1.16	1.05–1.29	0.0040	1.06	0.91–1.23	0.4825	1.11	0.99–1.24	0.0835	‐	‐	‐
Private	0.92	0.86–0.98	0.0125	0.88	0.80–0.96	0.0046	1.02	0.95–1.10	0.5275	‐	‐	‐
No Insurance	0.97	0.84–1.13	0.7546	1.89	0.69–1.12	0.3034	1.03	0.89–1.21	0.6632	‐	‐	‐
Unknown	0.87	0.77–0.98	0.0254	0.85	0.71–1.02	0.0780	0.94	0.83–1.08	0.4131	‐	‐	‐
Histology												
Clear cell (Ref)	‐	‐	‐	‐	‐	‐	‐	‐	‐	‐	‐	‐
Papillary	0.97	0.86–1.10	0.6669	0.91	0.79–1.06	0.2305	1.18	1.00–1.40	0.0473	1.18	1.00–1.39	0.0507
RCC NOS	1.33	1.25–1.43	<0.0001	1.19	1.08–1.32	0.0004	1.28	1.19–1.39	<0.0001	1.26	1.16–1.36	<0.0001
Chromophobe	0.58	0.45–0.76	<0.0001	0.53	0.40–0.71	<0.0001	0.87	0.62–1.23	0.4357	0.87	0.62–1.22	0.4181
RTCC	1.83	1.68–2.00	<0.0001	1.70	1.51–1.91	<0.0001	1.56	1.39–1.75	<0.0001	1.53	1.36–1.72	<0.0001
Others	2.13	1.94–2.34	<0.0001	1.95	1.69–2.25	<0.0001	1.90	1.73–2.10	<0.0001	1.87	1.70–2.10	<0.0001
Stage												
Localized (Ref)	‐	‐	‐	‐	‐	‐	‐	‐	‐	‐	‐	‐
Regional	3.13	2.90–3.39	<0.0001	3.0	2.7–3.2	<0.0001	‐	‐	‐	‐	‐	‐
Distant	9.15	8.45–9.90	<0.0001	‐	‐	‐	‐	‐	‐	‐	‐	‐
Unknown	1.73	1.54–1.94	<0.0001	‐	‐	‐	‐	‐	‐	‐	‐	‐
Surgery												
Yes (Ref)	‐	‐	‐	‐	‐	‐	‐	‐	‐	‐	‐	‐
No	2.58	2.41–2.76	<0.0001	3.17	2.87–3.51	<0.0001	2.14	1.99–2.30	<0.0001	2.13	1.98–2.30	<0.0001
Unknown	1.58	0.99–2.53	<0.0001	2.80	1.24–6.30	0.0129	1.61	0.95–2.74	0.0768	1.53	0.90–2.60	0.1136
Chemotherapy												
Yes (Ref)	‐	‐	‐	‐	‐	‐	‐	‐	‐	‐	‐	‐
No	1.00	0.93–1.08	0.93	0.59	0.51–0.68	<0.0001	1.35	1.26–1.44	<0.0001	1.34	1.25–1.43	<0.0001
Unknown	1.20	1.01–1.43	0.0392	0.92	0.64–1.33	0.6616	1.14	0.97–1.35	0.1078	1.15	0.98–1.36	0.0892
Radiation												
Yes (Ref)	‐	‐	‐	‐	‐	‐	‐	‐	‐	‐	‐	‐
No	0.88	0.80–0.97	0.0082	0.50	0.38–0.66	<0.0001	0.89	0.83–0.96	0.0037	0.90	0.83–0.97	0.0050
Unknown	0.77	0.55–1.10	0.1400	0.52	0.19–1.45	0.2133	0.89	0.65–1.20	0.4341	0.89	0.66–1.21	0.4677

Abbreviations: APIs, Asian and Pacific Islanders; CI, confidence interval; HR, hazard ratio; NHB, non‐Hispanic Black; NHW, non‐Hispanic White; RCC NOS, renal cell carcinoma not otherwise specified; RTCC, renal transitional cell carcinoma.

^a^ Adjusting for smoking status, age, histology, and treatment (surgery, chemotherapy, and radiation).

## 3 RESULTS

All primary KC cases diagnosed during 2005–2018 in Florida were analyzed, totaling 36,150; 63.2% were male and 36.8% were female. A majority of KC cases (51.2%) were either current or former smokers (Table 1).

Most KC patients were White (74.0%), followed by Hispanic (13.8%), Black (10.4%), API (0.9%), and American Indian (0.1%). The largest proportion of KC cases (30%) was diagnosed in those 65–74 years old. The median age for KC in never smokers (66 years) was not significantly different from KC in ever smokers (67 years) (*p* = 0.443). Among male KC patients, the proportion of ever smokers was 57.0% and among female KC patients 41.2% were ever smokers. Proportions of ever smokers varied by race/ethnicity with 54.7% in Whites, 43.6% in Blacks, and 39.4% in Hispanics. By insurance status, those under Medicaid were often (53.9%) ever smokers, followed by those under Medicare (53.1%) and those with private insurance (48.3%). In terms of histology, 62.6% of RTCC cases occurred among ever smokers. Among those diagnosed with chromophobe type, 38.8% were ever smokers. More than half of KC cases (64.9%) were diagnosed at a localized stage while 14.9% were diagnosed in regional stage and 14.6% in distant stage. The proportion of ever smokers was higher among those with distant stage (56.1%) followed by regional stage (53.5%) and localized stage (49.7%). Most patients received surgery (78.1%), while 8.2% received chemotherapy, and only 3.6% received radiation. Proportions of smokers also varied by primary tumor location: 50.3% of those with kidney cancer proper (renal cortex, C64.9) and 62.4% of those with renal pelvis cancer (C65.9) were ever smokers. Of note, these proportions are lower than those reported for lung cancer.^25^

Among all patients with KC, the median follow‐up time was 3.68 years, and 9996 (27.7%) deaths were due to KC recorded during 2005–2018, while 26,154 (72.3%) were alive at the end of the follow‐up or died of other causes. The age‐standardized five‐year KC specific survival for current smokers was 65.3% (95% CI: 64.1%–66.5%). For former smokers it was 70.6% (95% CI: 69.7%–71.5%) and for never smokers 75.3% (95% CI: 74.6%–76.0%) (*p* < 0.0001) (Figure 2).

The univariable Cox regression analysis showed that all socio‐demographic and clinical factors were statistically significant predictors of CSS (Table 2). In multivariable Cox regression (Table 2), former and current smokers had 14% (HR: 1.14, 95% CI: 1.10–1.20) and 30% (HR: 1.30, 95% CI: 1.23–1.40) greater risk of KC mortality, respectively, as compared to never smokers. The risk of KC mortality increased with age. Those in the age groups 65–74 and 75+ years had 2.12 (95% CI: 1.87–2.40) and 2.92 (95% CI: 2.57–3.32) times the risk of KC mortality, respectively, compared to those aged 15–44 years. Hispanics had a lower risk of KC mortality as compared to Whites (HR: 0.82, 95% CI: 0.77–0.87), while no statistical differences in survival were observed between Whites and Blacks. Those with low socioeconomic status had 7% higher risk of KC mortality (HR: 1.07, 95% CI: 1.00–1.14; HR), compared to those with high socioeconomic status. Patients with Medicaid had 15% higher risk of death due to KC (HR: 1.15, 95% CI: 1.06–1.24), compared to those with Medicare. KC patients with chromophobe histology type had a lower risk (HR: 0.61, 95% CI: 0.51–0.72), and with RTCC, a greater (HR: 1.80, 95% CI: 1.69–1.93) risk of dying from KC, compared to those with clear cell. With more advanced stages of cancer, the risk of KC mortality escalated: regional stage with more than 3‐fold risk (HR: 3.44, 95% CI: 3.24–3.65) and distant stage at nearly 10 times higher risk of death (HR: 9.91, 95% CI: 9.33–10.53), compared to those in localized stage.

In a direct comparison of current and former smokers in the multivariable analysis, current smokers had 13% greater risk of dying due to KC as compared to former smokers (HR: 1.13, 95% CI: 1.06–1.20, *p* < 0.0001) (Table 3, Model 1). When restricted to localized‐ and regional‐stage KC (Table 3, Model 2), current smokers maintained a greater risk of KC mortality as compared to former smokers (HR: 1.21, 95% CI: 1.12–1.32, *p* < 0.0001). Similarly, in the multivariable analysis restricted to distant staged KC (Table 3, Model 4), after controlling for significant predictors from Model 3, current smokers had a higher risk of KC mortality, compared to former smokers (HR: 1.10, 95% CI: 1.01–1.20, *p* = 0.0284).

## 4 DISCUSSION

This unique registry‐based study, utilizing all KC cases in Florida during 2005–2018, examines the association between cigarette smoking status at diagnosis and KC survival and reports two key findings. Firstly, smoking at diagnosis is an independent predictor of cancer‐specific survival. The risk of KC death was elevated for both current and former smokers in relation to never smokers, but more importantly, current smokers had a 13% greater risk of KC mortality, compared to former smokers when controlling for other factors. Secondly, while other studies had established a negative prognostic effect for smoking at diagnosis restricted to metastatic KC,^11^ our study extends these findings to patients diagnosed in localized and regional stages of the disease, with current smokers at 21% higher risk of death compared to former smokers, adjusting for other factors.

In past studies researchers have found, for distant disease only, an increased risk of overall and cause‐specific KC mortality among current smokers.^15^ Another study by Parker et al. showed a potential association between current smoking and worse survival, though, the relationship disappeared after adjustment.^26^ Lastly, a 2000 study conducted among a small sample of 148 clear cell RCC cases at Brigham and Women's Hospital also failed to establish a significant association between smoking status and survival outcome after adjustment.^16^

The biological mechanism underlying the association between cigarette smoking and poor KC prognosis may involve the toxic effect of nicotine leading to endothelial cell dysfunction and abnormal hemodynamic changes. This causes DNA damage, neoplastic cell proliferation, cancer progression, as well as, resistance to chemotherapy and radiation.^27^ Mutations in the p53 gene have also been attributed to the effect of polycyclic aromatic hydrocarbons present in tobacco.^28^ In 2012, Kroeger et al. found significantly higher mutated p53 expression among current smokers.^29^ Immune suppression, mediated by declining T‐cell and natural killer cells and leading to the facilitation of tumor growth, has also been associated with cigarette smoking.^30^ In a recent molecular study, Huang et al. exposed cancerous renal cells in a dose‐dependent manner for 4 months to specific nitrosamine 4‐(methylnitrosamino)‐1‐(3‐pyridyl) ‐1‐butanone (NNK, nicotine‐derived nitrosamine), the major and the most potent carcinogen among nicotine‐derived nitrosamines, found in elevated levels in cigarette smoke and among smokers. Their results showed that NNK promoted cancerous renal cell growth and migration.^31^

In addition to the prognostic effect of smoking, other differences were found, mostly in agreement with previous studies. Hispanics held a survival advantage in relation to Whites; by histology, chromophobe type had a better prognosis compared to all other types, while renal cell carcinoma not otherwise specified (RCC NOS) and RTCC showed a disadvantage in survival in relation to other groups. The better survival outcomes of chromophobe cell KC were consistent with the existing aggregate literature.^32^, ^33^ Unfortunately, RCC NOS description is a loose histological term that may refer to clear cell, papillary, or chromophobe as well as other subtypes, and thus, it is not possible, in this study just like in previous studies, to fully characterize the survival of specific histological groups.^34^ In the analysis restricted to KC in localized and regional stages papillary and chromophobe histology type showed better survival outcomes as compared to the clear cell type in agreement with previous studies,^35^, ^36^ a difference which disappears if KC was diagnosed in distant stage. Lastly, patients on Medicaid had the worst survival outcomes of all insurance types, even after adjustment for socioeconomic status, also consistent with previous research.^37^

A major strength of this study is the use of all‐inclusive, racially, and ethnically diverse, state‐level data consisting of patients receiving care from a myriad of distinct healthcare facilities and presenting with various insurance statuses. Previous studies on this topic are primarily hospital or cohort‐based studies, in which there is potential for selection bias associated with referral patterns and willingness to participate, often resulting in a primarily Caucasian sample population, perpetuating limited generalizability of the findings for other marginalized populations. Furthermore, existing studies on smoking as a prognostic factor have been based on small samples, restricted to metastatic renal cell carcinoma treated with CNT, and limited to overall survival, thus including causes of death other than KC.^11^, ^14^, ^38^ Instead, this study uses cause‐specific survival as the outcome, limiting the mortality impact of comorbidities, and includes both metastatic and non‐metastatic KC cases. Not without limitations, our study lacks information regarding the dose and duration of smoking, such as pack‐years, and the duration of smoking cessation among former smokers. Additionally, the effects of different surgical, chemotherapy and radiation treatment options on KC survival were not assessed. The apparently negative impact of radiotherapy and chemotherapy in some of the models is related to their indication for treatment occurring in more advanced stages of the disease. By race‐ethnicity, cancer registry data generally overestimate the survival of foreign‐born populations, especially Hispanics and Asians,^39^ which could have affected our results. Possible covariates associated with KC prognosis such as body mass index, alcohol consumption, and other comorbidities were not available for analysis, and differences in type and severity of tobacco‐related comorbidities between past versus current smokers were unknown.

In conclusion, this study highlights that smoking is an independent prognostic factor of KC survival, regardless of the stage at diagnosis. Current smokers, followed by former smokers, had a higher risk of KC mortality, as compared to never smokers in all stages of KC. Our findings can help clinicians identify patients who are at a higher risk of kidney cancer mortality and who may benefit from smoking cessation in addition to the recommended treatment strategies. Smoking cessation programs and counseling may be an important component of clinical management of KC patients and more successful outcomes. In addition, our study suggests that further research could focus on the impact of smoking cessation on clinical KC outcomes other than death such as recurrence, but also survivorship outcomes such as quality of life. The study also highlights the importance of addressing health disparities in kidney cancer outcomes. Patients on Medicaid have the worst survival outcomes of all insurance types, even after adjustment for socioeconomic status. Clinicians and researchers should consider interventions that address these disparities, such as improving access to healthcare and ensuring that all patients receive optimal treatment regardless of their insurance status. Further prospective studies are warranted to quantify this type of association, assess the role of distinct types of tobacco, and investigate the role of smoking cessation on survival outcomes of KC patients.

## AUTHOR CONTRIBUTIONS

**Amrit Baral:** Conceptualization (lead); formal analysis (lead); investigation (lead); methodology (equal); project administration (lead); visualization (equal); writing – original draft (lead); writing – review and editing (equal). **Hannah M. Cranford:** Visualization (supporting); writing – review and editing (equal). **Janaki Sharma:** Resources (equal); supervision (equal); validation (equal); writing – review and editing (supporting). **Paulo S. Pinheiro:** Conceptualization (equal); data curation (lead); formal analysis (supporting); funding acquisition (lead); investigation (equal); methodology (equal); project administration (equal); resources (equal); software (equal); supervision (lead); validation (equal); writing – review and editing (equal).

## FUNDING INFORMATION

This work was partially supported by the Bankhead Coley Research Program of the State of Florida, Grant Number 20B16. This research was also supported by the National Cancer Institute Award Number P30CA240139.

## CONFLICT OF INTEREST STATEMENT

The authors declare no conflicts of interest.

## Data Availability

Data Availability Statement: The datasets used in this study are available by request with required approvals from the Florida DOH Cancer Registry Program and Institutional Review Board. Applications.
